# Integration of miRNAs, Degradome, and Transcriptome Omics Uncovers a Complex Regulatory Network and Provides Insights Into Lipid and Fatty Acid Synthesis During Sesame Seed Development

**DOI:** 10.3389/fpls.2021.709197

**Published:** 2021-07-29

**Authors:** Yin-Ping Zhang, Yuan-Yuan Zhang, Kiran Thakur, Fan Zhang, Fei Hu, Jian-Guo Zhang, Peng-Cheng Wei, Zhao-Jun Wei

**Affiliations:** ^1^Anhui Academy of Agricultural Sciences, Crop Research Institute, Hefei, China; ^2^School of Food and Biological Engineering, Hefei University of Technology, Hefei, China; ^3^College of Agronomy, Anhui Agricultural University, Hefei, China; ^4^Key Laboratory of Rice Genetic Breeding of Anhui Province, Rice Research Institute, Anhui Academy of Agricultural Sciences, Hefei, China

**Keywords:** sesame, lipid fatty acid, biosynthesis, network biology, seed development

## Abstract

Sesame (*Sesamum indicum* L.) has always been known as a health-promoting oilseed crop because of its nutrient-rich oil. In recent years, studies have focused on lipid and fatty acid (FA) biosynthesis in various plants by high-throughput sequencing. Here, we integrated transcriptomics, small RNAs, and the degradome to establish a comprehensive reserve intensive on key regulatory micro RNA (miRNA)-targeting circuits to better understand the transcriptional and translational regulation of the oil biosynthesis mechanism in sesame seed development. Deep sequencing was performed to differentially express 220 miRNAs, including 65 novel miRNAs, in different developmental periods of seeds. GO and integrated KEGG analysis revealed 32 pairs of miRNA targets with negatively correlated expression profiles, of which 12 miRNA-target pairs were further confirmed by RT-PCR. In addition, a regulatory co-expression network was constructed based on the differentially expressed gene (DEG) profiles. The *FAD2*, *LOC10515945*, *LOC105161564*, and *LOC105162196* genes were clustered into groups that regulate the accumulation of unsaturated fatty acid (UFA) biosynthesis. The results provide a unique advanced molecular platform for the study of lipid and FA biosynthesis, and this study may serve as a new theoretical reference to obtain increased levels of UFA from higher-quality sesame seed cultivars and other plants.

## Introduction

Sesame (*Sesamum indicum* L.), as one of the oldest herbaceous diploid oilseed plants (2n = 26) in the Pedaliaceae family with a genome size of approximately 369 Mb, is mainly cultivated in tropical and subtropical regions of Asia, Africa, and South America (Wei X. et al., 2016; Majdalawieh et al., 2020). Among the major cultivated oil crops worldwide, sesame as a commercial material contains the highest number of fatty acids (FAs) (Wacal et al., 2019; Zhang et al., 2020), and among the FA types, linoleic, oleic, palmitic, and stearic acids are the main components that make up the total FA configuration of sesame oil (Elleuch et al., 2007; Adebowale et al., 2011; Bhunia et al., 2015). In previous studies, unsaturated fatty acids (UFAs) are considered as a quality indicator of edible oils in the core area of research for the development of functional and industrial products (Wang et al., 2016; Ahmed et al., 2020), which can prevent cardiovascular sclerosis and reduce blood pressure and blood lipids (Li et al., 2015). Therefore, increasing the UFAs content is a conventional way to improve the quality of sesame varieties and increase the seed oil content, which are also identified as the most important strategies to meet the current needs of consumers (Wang D.D. et al., 2018).

With the advent of high-throughput sequencing, genome breeding has developed rapidly, such as quantitative genetics, genetic breeding, genetic relatedness, and diversity (Wei X. et al., 2016; Yan et al., 2016; Kumar et al., 2017). In recent years, although the whole transcriptome sequencing of sesame has been described, information on the role of micro RNAs (miRNAs) and target genes, especially miRNA expression profiles involved in lipid and FA biosynthesis of sesame oil, is still poorly understood. miRNAs as endogenous non-coding RNAs (18–26 nt) regulate gene expression through post-transcriptional translational inhibition or direct cleavage of transcripts based on complementarity between miRNA and its target gene (La Sala et al., 2018; Sevgi, 2018). Recent studies have demonstrated the role of miRNAs in almost all life activities, such as biosynthesis, cell reproduction, plant resistance to biotic and abiotic stresses, and metabolic pathways (Han et al., 2016; Zhao et al., 2018, 2020; Zheng et al., 2019). In banana fruits, 128 known miRNAs belonging to 42 miRNA families were determined, and 12 new miRNAs were identified; in olive, 135 conserved miRNAs were identified and 38 putative new miRNAs were discovered; in strawberry, a total of 88 known miRNAs and 103 targets cleaved by 19 known miRNAs families were discovered (Xu et al., 2013; Yanik et al., 2013; Dan et al., 2018). Notably, from 18 sesame libraries, 351 known and 91 novel miRNAs, 116 miRNAs were reported to regulate salt stress response (Zhang et al., 2020). Similarly, major miRNA-target pairs and the complex regulatory network responsible for terpenoid biosynthesis in tea leaves (Zhao et al., 2018) and cadmium phytoremediation in hyperaccumulator *Sedum alfredii* were revealed by integrating small RNAs, degradome, and transcriptomics (Han et al., 2016). Subsequently, the lipid biosynthetic pathway and key periods of FA biogenesis and associated proteins were deciphered in the developing endosperm of *Cocos nucifera* (Reynolds et al., 2019) and tree peony seeds (Yin et al., 2018), respectively. Similarly, regulatory networks of lipid and FA metabolism were revealed in *Carya cathayensis* Sarg (Huang et al., 2016, 2021) and oil palm (Zheng et al., 2019) by transcriptomic and miRNA sequencing, respectively.

From the previous studies, it can be speculated that the integration of transcriptomic and lipidomic studies can provide a comprehensive view of the dynamic processes regulating the seed development and oil biosynthesis in various plants (Dossa et al., 2017; Wang et al., 2019; Chen et al., 2020). However, no such data have been collected for sesame seeds to date. Although there is sparse information on the chemical compositions (lignan, sesamin, and sesamolin), amino acid types and contents, and FA properties of sesame seeds from different regions, so far no transcriptome and degradome analyzes have been performed (Wei L. B. et al., 2016; Gacek et al., 2017; Sui et al., 2018; Ahmed et al., 2020). The specific functional genes and key metabolic pathways that regulate the synthesis of UFA in sesame seeds in particular are still unknown and unexplored. Therefore, a more comprehensive study on the controlling roles of miRNAs and their targets in lipid and FA biosynthesis is needed to further elucidate the underlying mechanism in the differential seed development of sesame.

Herein, the FA compositions in developing sesame seeds were analyzed, and the first-hand data on the combination of transcriptomics, small RNAs, and degradome in sesame seeds harvested in different developmental periods were generated and integrated to develop a comprehensive resource. We anticipate that this study will provide valuable information on the mechanism of lipid and FA biosynthesis in sesame seeds in different developmental periods. Furthermore, this study would promote molecular breeding research to improve the value-adding effects of sesame oil and the resulting functional plant products. Moreover, the collected miRNA expression patterns and extensive miRNA-mRNA regulatory network data can also be used for the upcoming expansion of plant oils with enriched UFA content.

## Materials and Methods

### Chemicals, Plant Materials, and Total RNA Extraction

For this study, sesame seeds named Wan Zhi No.2 (WZ2) were collected from the experimental field of the Crop Research Institute of Anhui Academy of Agricultural Sciences, Hefei, China in 2019. The whole growth period of WZ2 was about 88 days, and its seed yield was recorded at 1,788 kg/ha with a crude fat content of 55% (Wang et al., 2011). It was bred from sexual hybridization and systematic breeding of a local variety “*Xiaozibai*” (female parent) and *Yuzhi No.4* (male parent). Beginning at 7 days after flowering (DAF), three biological replicates of sesame seeds for each sampling development period (7, 14, 21, and 28 DAF in August 2019) were handpicked until fully matured and then stored in liquid nitrogen at −80°C until further use.

For transcriptomic analysis, miRNA sequencing, and degradome analysis, total RNA of each sample was extracted from the collected seeds, including three biological replicates, using Trizol reagent (Invitrogen, Waltham, MA, United States) according to the previous method (Liu et al., 2016).

### GC-MS Analysis of FA in Sesame Seed Oil

After drying and crushing, the sesame oil was extracted using the Soxhlet extraction method, and the solvent was evaporated with a rotary evaporator (Dbrowski et al., 2020). The FA composition of the sesame oil was analyzed by gas chromatography-mass spectrometry (Agilent GC-MS 7890A, Agilent Technologies, Santa Clara, CA, United States) equipped with a DB-5MS column and NIST 08 (Gaithersburg, MD, United States) according to the procedure described (Zhu et al., 2021). Crude fat 50 mg was weighed into a 10-ml centrifuge tube followed by the addition of 2 ml 1% H_2_SO_4_-CH_3_OH reagent and heating for 30 min at 70°C in a water bath. Then, 2 ml of N-hexane was added, and water was filled to the top of the centrifuge tube (10 ml). The whole mixture was shaken well and allowed to stand for 24 h. After the centrifugation, supernatant was analyzed by GC-MS. GC conditions were as follow: DB-5 column (30 mm × 0.32 mm, 0.25 μm), the carrier gas was high purity helium, the injection temperature was 250°C, the column flow rate was 1.25 ml/min, and the micro injector was used to inject 1.0 μl. The temperature rise program was 50°C (2 min), 5°C/min to 270°C. Mass spectrometry conditions were as follow: EI ion source, ion source temperature 220°C, GC-MS interface temperature 250°C, electron energy 70 eV, scan mass range 60–500 m/z.

According to the results of the GC-MS analysis, the percentage of each FA component was calculated by the peak area normalization method (i.e., the percentage of each peak area to the total peak area).

### Transcriptome Sequencing and Assembly Analysis

A cDNA library constructed from the pooled RNA from the different samples of sesame seeds was sequenced using the Illumina 6000 sequencing platform according to Illumina paired-end RNA-seq. Prior to assembly, the low-quality reads were omitted, and overall cleaned paired-end reads were generated. The raw sequence data were submitted to the NCBI database, and the reads of the samples were mapped to the UCSC^1^ reference genome^2^ using the HISAT package. Then, all transcriptomes were generated using StringTie to examine the expression level for FPKM after the final transcriptome was constructed. Here, the differentially expressed genes (DEGs) and mRNAs with *p* value of less than 0.05 and a log_2_| fold change| of more than 1 were screened with the R package (Lyu et al., 2020).

### Small RNA Sequencing and miRNA Identification

In this assay, the 12 small RNA libraries (three biological replicates) of the four different period samples were constructed and processed by screening with a high-throughput sequencing method (Illumina HiSeq 2500 platform) at LC-BIO (Hangzhou, China). Raw RNA reads were generated and estimated using Illumina FastQC to obtain Q30 data. The raw reads were subjected to an in-house program, ACGT101-miR (LC Sciences, Houston, TX, United States) to remove adapter dimers, junk data, low complexity reads, common RNA families (rRNA, tRNA, snRNA, and snoRNA) and repeats, and the valid reads were obtained. Four samples of small RNA sequencing were analyzed based on the previous bioinformatics method (Yuan et al., 2013; Bisgin et al., 2018). Read distribution was calculated for different libraries and normalized for miRNA prediction using global normalization methods (Lei et al., 2021; Zhang et al., 2021).

The differentially expressed miRNAs in different seed development periods were evaluated using the DESeq R package (1.8.3) and found to be significant at *P* < 0.05. Then, to identify the differentially expressed known and novel miRNAs in sesame seeds, unique sequences with length of 18--25 nt were selected to map the specific species precursors in miRBase^3^ by BLAST search. The unique sequences that can be further compared with the genome were considered as known miRNAs. The unique sequences that can be compared with the genome but cannot be compared with the pre-miRNAs of the species selected in miRBase were assumed to be novel miRNA candidates.

### Degradome Analysis and Target Identification

The RNA libraries containing three biological replicates from four samples of the developmental period of sesame seeds were mixed as one degradome library. Then, the four small RNA samples and one degradome composite sample were analyzed on the sequencing platform (IlluminaHiSeq 2500) at LC-Bio Co., Ltd., (Hangzhou, China), and the degradome libraries were constructed according to the description of the manufacturer. Furthermore, the degradome reads were aligned with the transcriptome data of the sesame samples. Finally, all the identified known and novel miRNA-target genes were mapped to the database (see text footnote 2). Analysis of Gene Ontology (GO) and Kyoto Encyclopedia of Genes and Genomes (KEGG) enrichment was performed using Agrigo and the Perl script, respectively, and the non-redundant GO enrichment terms were visualized using Revigo. In this study, a miRNA target gene network was constructed using Cytoscape (version 3.4.0).

### Analysis of Differentially Expressed Target Genes

To obtain the target genes of lipid and FA synthesis, 12 RNA libraries were constructed from the sesame seed samples collected in four different developmental periods. For each library, all sequences were mapped to the CDS, and the number of each gene was counted using standard parameters. All the clean reads were mapped to the assembled unigenes of *S. indicum* L. for annotation. The RPKM of each gene referred to the number of sequencing fragments mapped to the CDS of the gene and was calculated by one thousand transcripts per million sequencing bases. According to the similarity of the gene expression profiles of samples, the heat map of the clustering expression pattern was constructed using log_10_ (FPKM + 1) to represent the miRNAs and gene expression. The standard for measuring the DEGs among the different samples was based on *P-value* < 0.05 and fold change greater than 2.

### Co-expression Analysis

For further exploration of the relationship between each module and FA synthesis, DEGs and expression levels of each module were screened, and the correlation between each module and nine types of FA synthesis was tested. Weighted co-expression network analysis was performed using the three elements (node, edge line, and network) of Cytoscape network graph (LC-Bio Co., Ltd., Hangzhou, China). As a result of the data collection and import with the Cytoscape package, the basic framework of the network graph was created after confirmation. All the other parameters were used with modification and adjustment. The co-expression networks for selected miRNAs and target pairs were visualized using Cytoscape (version 3.4.0).

### Analysis of Gene Expression and Validation by qRT-PCR

For data validation, we selected 12 differentially expressed miRNA-target pairs to perform qRT-PCR to verify the high-throughput sequencing results. SYBRPrimeScript^TM^ miRNA RT-PCR Kit (TaKaRa, Dalian, China) was used for reverse transcription reactions for miRNAs, and PrimeScript RT Reagent Kit (TaKaRa, Dalian, China) was used for reverse transcription of cDNAs. U6 and beta-tubulin (TUB) were selected for normalization of miRNAs and target genes, respectively (Zhang et al., 2020). The names of miRNAs and their target genes, sequences, and primers in RT-qPCR test experiment are given in Supplementary File 1. The 2^–Δ^ ^Δ^ ^*Ct*^ method was used to calculate the relative fold change of miRNA target genes, and the PCR reaction of each sample was repeated three times.

## Results

### Lipid Accumulation in Sesame Seed Development

**FIGURE 1:**
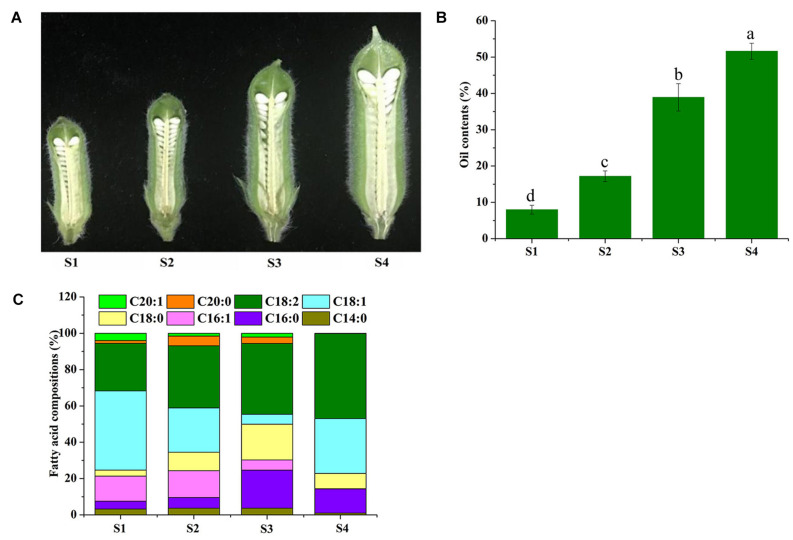
Phenotypic observation and fatty acid determination during sesame seed development. The developmental process of sesame seeds (S1–S4) **(A)**. Collection time was at 7, 14, 21DAF, and until 28 days after flowering (DAF) (the time point of seed maturity). **(B)** The oil content percent of sesame seed at four time points. **(C)** The percent of different fatty acid compositions at four time points of sesame seed.

In this study, we harvested sesame seed samples of the four developmental periods to evaluate their FA composition by gas chromatography-mass spectrometry (GC-MS) (Figure 1). The results show five dominant components, namely, oleic acid (C18:1, 43.61% of total FAs at S1), linoleic acid (C18:2, 46.77% of total FAs at S4), palmitic acid (C16:0, 20.96% of total FAs at S3), *cis*-7-hexadecenoic acid (C16:1, 14.72% of total FAs at S2), and stearic acid (C18:0, 19.7% of total FAs at S3) as shown in Figure 1B. It is found that all these five FAs had higher percentage and dominated over the other FAs (more than 89% of the total FA) during the four consecutive periods and reached 98.8% of the total FA in the S4 periods. Based on this, the development of sesame seeds can be divided into three distinct periods; starting with a low content of oil and FAs in the initial stage (S1-S2), followed by a rapid accumulation of oil in the second and third stages (S3), and a gradual increase in oil and FAs until full maturation (S4) (Figures 1B,C). However, saturated fatty acids (SFAs) showed a slight decrease, and UFAs showed an increase at S4; in particular, oleic acid, linoleic acid, and hexadecenoic acid was the dominant factors to this significant increase (Figure 1C). Overall, oleic acid and linoleic acid were maintained at relatively high levels during sesame seed development, and UFAs dominated total FAs with relatively high proportions (Figure 1C).

### Transcriptome Sequence Analysis in Sesame Seeds Under Different Growing Time Points

RNA pools were obtained from sesame seed samples harvested at four different developmental time points to construct the transcriptome library. After refining the raw sequence data, only 147,561,222 reads remained valid out of 213,772,171 raw reads. Table 1 shows the annotation of sequence alignments uniq_miRNAs with the NCBI non-redundant protein (Nr) database. In addition, the results of the BLASTX algorithm showed the presence of 6,292 uniq_miRNAs with an average length of 70b (Table 1). Twelve RNA sequencing data with three biological replicates from the four different developmental periods were accumulated for the identification of DEGs with the aim of gene expression profile.

**TABLE 1:** Data analysis of transcriptome sequencing for *Sesamum indicum* L.

**Group**	**S1_Seed**	**S2_Seed**	**S3_Seed**	**S4_Seed**
Raw reads	41,739,967	38,964,110	60,033,649	73,034,445
Valid reads	32,450,409	28,931,109	40,219,897	45,959,807
Number of uniq_miRNAs	1,427	1,278	1,833	1,754
Average Uniq_miRNA length (bp)	70	70	69	69
Q20 (%)	98	98	98	98
GC (%)	52	53	52	52

### Small RNA Sequencing Profile

**FIGURE 2:**
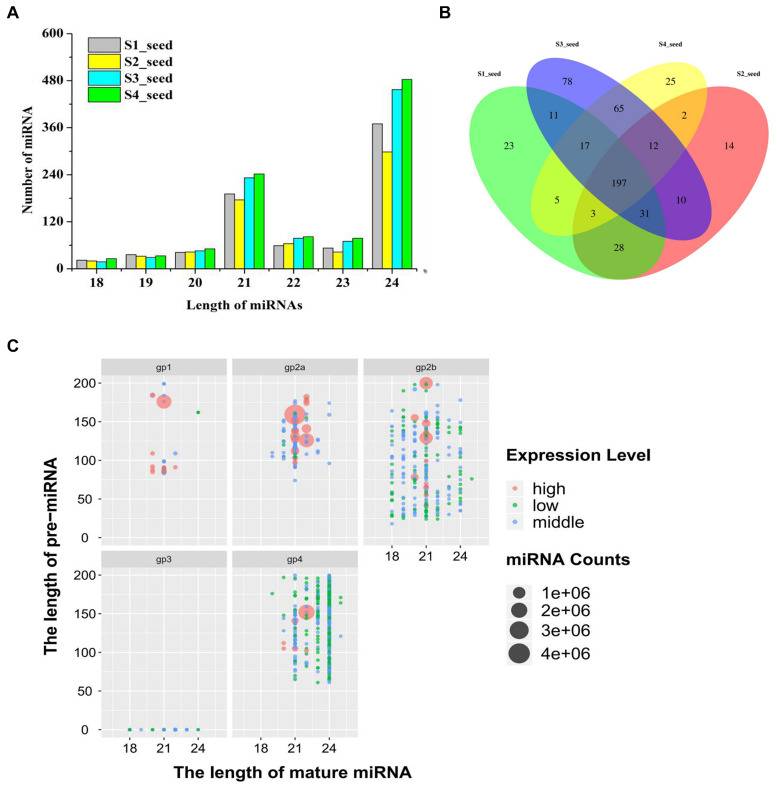
**(A)** Number of different-length miRNA distribution in different development periods of sesame seed, **(B)** Venn diagrams of differentially expressed miRNAs in different development periods of sesame seed, and **(C)** length of miRNA distribution of five groups for expression level (high-level expression over average; middle-level expression >10 but less than average; low-level expression < 10).

Small RNA sequencing for 12 libraries was performed to decipher miRNA regulated post-transcriptional changes associated with lipid and FA biosynthesis. Figure 2A depicts the size distributions and similar patterns of unique and valid 18- to 25-nt-long sequences obtained after applying the different steps of data processing. Among the 12 libraries, the 24-nt (49.1%) RNAs were found to be most abundant and diverse, followed by 23% small RNAs with 21-nt-long sequences (Figure 2A and Supplementary File 2).

Based on the abundance in miRNA expression levels, 1,025 miRNAs were obtained and categorized into five different groups after database and sequence reading analysis (Figure 2C and Supplementary File 3). Among the unique mature miRNAs and pre-miRNAs, 354 unique mature miRNAs were categorized into groups 1, −2, and −3 (gp1, −2, and −3) (Supplementary File 3). In addition, the miRNAs from gp4 were not registered in miRBase, and 671 unique mature miRNAs corresponding to 664 pre-miRNAs were considered as new candidate miRNAs (Supplementary File 3). Interestingly, the pattern of the proportion of mature miRNAs in each sampling developmental period showed some similarity (Figure 2A). Moreover, a total of 220 miRNAs (114 known miRNAs and 106 novel miRNAs) were expressed in all four developmental periods, as shown in Figure 2B. In addition, the sum of the first nucleotide bases of miRNA was different between the known and novel miRNAs. Adenosine (A, 49.82%) was reported as the most abundant nucleotide, followed by uracil (U, 20.16%) and cytosine (C, 18.33%) (Supplementary File 4).

### Differential Expression of miRNAs in Sesame Seeds Under Different Growing Time Points

**FIGURE 3:**
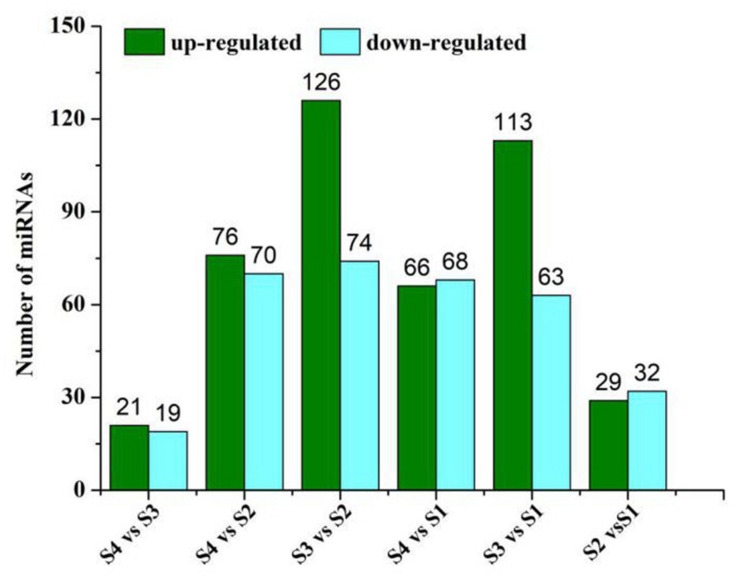
Number of up- and down-regulated miRNAs in different development periods of sesame seed.

We used the high-throughput sequencing method to analyze and compare the differential expression of miRNAs (DEmiRNAs) related to lipid and FA biosynthesis in four samples of sesame seeds. According to the analysis, among the 220 DEmiRNAs, 126 miRNAs were up-regulated and 74 miRNAs were significantly down-regulated in S3 vs. S2 (Figure 3). Moreover, the distribution patterns showed significant up-regulation of 113 miRNAs and down-regulation of 63 in S3 vs. S1 (Figure 3). These data suggest that stage 3 may be the critical control point that strongly influences miRNA expression levels during lipid and FA biosynthesis. According to the heat map of potential DEmiRNAs, 220 miRNAs were found to be significantly expressed across the four periods with similar distribution patterns of high or low expression levels verified after clustering analysis (Supplementary Figure 1).

### Prediction of Known and Novel miRNAs

**FIGURE 4:**
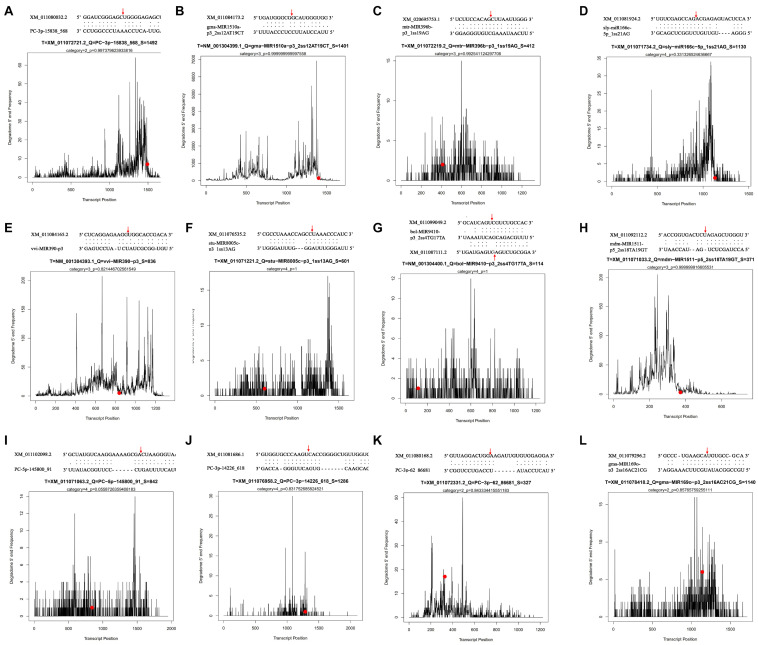
Some differentially expressed miRNA t-plots and cleavage sites identified by degradome sequencing.

To further understand the possible function and the regulatory patterns of the identified miRNAs and their target genes responsible for lipid and FA biosynthesis during sesame seed development, degradome sequencing was performed. According to the sequencing results, 33,155,150 (83.46% of the clean reads) refined reads were mapped to the 7,419,022 unigenes (70.84% of the input cDNA sequences) of sesame seeds (Supplementary File 5). From the mixed degradome data, 13,801 miRNAs with 1,006 targets were identified, including 65 novel miRNAs with 8,051 transcripts (Supplementary File 6). Among the referred 13,801 miRNAs, most miRNAs (804) could cleave six or more different transcript targets, while 202 miRNAs may have cleaved only few transcript targets (*t* ≤ 5 in number) (Supplementary File 6). Based on the in-depth analysis, the miRNA (aly-MIR408-p3_2ss18CT19TG) was identified with 520 targets, which is the highest number of transcripts cleavages by the same miRNA (Supplementary File 6). To summarize, 12 miRNAs were associated with the regulation of lipid and FA biosynthesis during the developmental periods of sesame seed. Among the 12 miRNAs, 11 could cleave only one transcript target, while the miRNA (*bol-MIR9410-p3_2ss4TG17TA*) cleaved two transcript targets (*XM_011087111.2* and *XM_011099049.2*), and the miRNA cleavage sites are shown in Figure 4.

### GO and KEGG Pathway Analyzes of Targets

**FIGURE 5:**
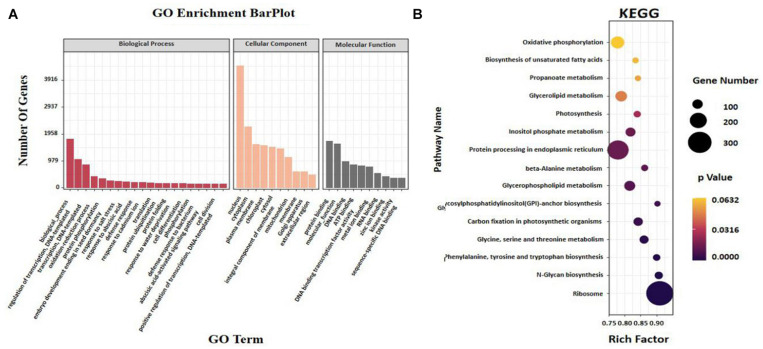
Analysis of **(A)** enriched GO and **(B)** KEGG of miRNAs and target genes in different seed development periods of sesame.

For the manifestation of the potential biological role of 2,296 miRNAs in sesame seed development, 2,186 target genes were categorized according to GO enrichment (Supplementary Figure 2). The enrichment data showed that most of the target genes were associated with biological processes involving “regulation of transcription,” “oxidation–reduction process,” and “protein phosphorylation” (Figure 5A). The GO terms “nucleus,” “cytoplasm,” and “plasma membrane” in the “cellular component” category accumulated the most frequent targets (Figure 5A). The GO terms “protein binding” and “molecular function” in the molecular function category had the highest number of targets compared with the other terms (Figure 5A). There, interesting GO terms, such as “embryo development ending in seed dormancy” and “abscisic acid-activated signaling pathway” were revealed (Figure 5A). For pathway analysis, KEGG annotations were performed in the top 20 pathways according to the KEGG annotated gene number. Most target genes were found enriched in “ribosome” and “protein processing in endoplasmic reticulum,” followed by “glycerolipid metabolism” and “glycerophospholipid metabolism” (Figure 5B). In addition, more genes were involved in “biosynthesis of secondary metabolites” consisting of “glycosylphosphatidylinositol (GPI)-anchor biosynthesis” and “biosynthesis of UFAs” (Figure 5B). Based on the enrichment data, “fatty acid degradation,” “beta-alanine,” and “inositol phosphate metabolism” were shown to be the most significantly enriched pathways (FDR 0.05) (Figure 5B).

### miRNAs and Target Genes in the Context of Lipid and Fatty Acid Biosynthesis

**FIGURE 6:**
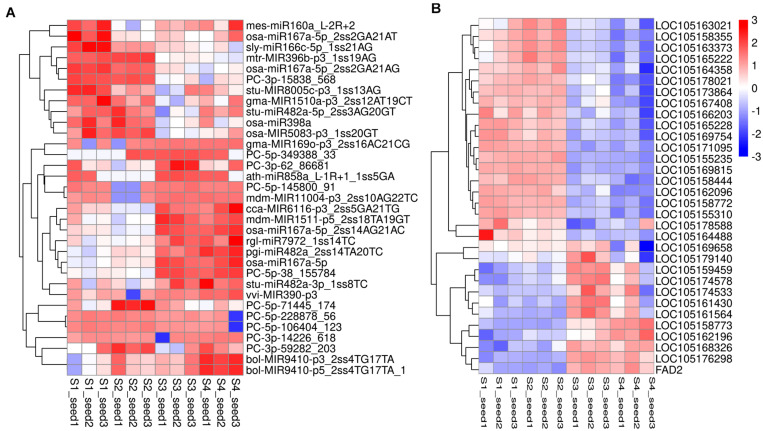
Heat map of DEmiRNAs **(A)** and their target genes **(B)** indicating the expression pattern in different seed development periods of sesame. The corresponding color changes from blue to red with signal intensity ranges from –3.0 to +3.0. The miRNAs expression values were standardized by *Z*-score standardization.

**FIGURE 7:**
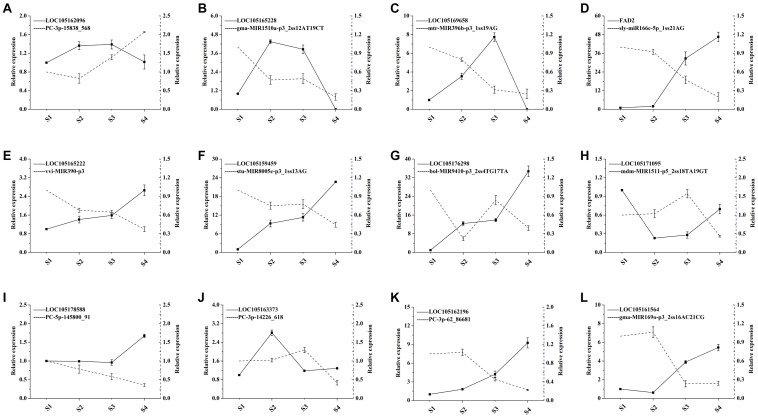
qRT-PCR validation of miRNAs and their target expression correlation in different development periods of sesame seed (*n* = 3). The dotted and solid lines express miRNAs and target genes, respectively.

The transcriptomics investigation revealed that a total of 220 DEmiRNAs corresponding to 3,705 target genes were also differentially expressed. After miRNA-target pair analysis, 32 miRNAs with 33 target genes were found to be associated with lipid biosynthesis in sesame seeds (Supplementary File 7). In this analysis, most DEmiRNAs and their targets showed a similar expression pattern as in high-throughput sequencing (Figure 6). According to the experimental qRT-PCR data, eight known miRNAs and their target pairs showed negative expression correlation (Figure 7). For example, miRNA (*syl-miR166c-5p-1ss21AG*) showed higher expression in S1 and S2, which decreased in S3 and S4, while target *FAD2* showed lower expression in S1 and S2, which increased in S3 and S4. Moreover, four novel miRNA-target pairs (*PC -3p-15838_568/LOC105162096*, *PC -5p-145800_91/LOC105178588*, *PC -3p-62_86681/LOC105162196*, and *PC -3p-14226_618/LOC105163373*) also showed inverse association at the expression level, which control transcriptional repression by targets and their corresponding miRNAs (Figure 7). In addition, we detected three miRNAs-target pairs (*sly-miR166c-5p_1ss21AG/FAD2*, *stu-MIR8005c-p3_1ss13AG/LOC105159459*, and *gma-MIR169o-p3_2ss16AC21CG/LOC105161564*) that exhibited not only a relationship at the high expression level but also an apparent negative relationship. Interestingly, the miRNA *bol-MIR9410-p3_2ss4TG17TA* was found to have two target genes (*LOC105176298* and *LOC105167408*). We hypothesize that the functional analysis and cloning of the three genes *FAD2*, *LOC105159459*, and *LOC105161564* could potentially facilitate the deciphering of the mechanistic details associated with the biosynthesis of UFAs during sesame seed development.

### Gene Co-expression Network Associated With Lipid and Fatty Acid Biosynthesis

**FIGURE 8:**
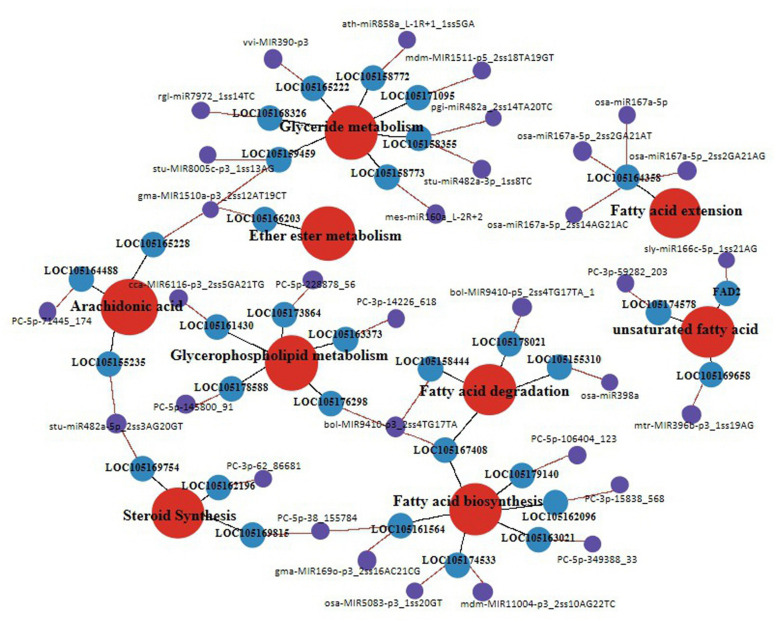
Schemata of lipid synthesis enzymes and genes that related to fatty acid metabolism pathway in the different development periods of sesame seeds. The red color circles show different lipid biosynthesis pathways, blue colors show different expression genes, and purple color shows different expression miRNAs.

To elucidate the function of miRNA-target pairs in the context of lipid and FA biosynthesis regulation in different developmental periods, we constructed a coexpression network with 33 genes from nine categories, mainly associated with glycerolipid metabolism, glycerophospholipid metabolism, UFAs, FA biosynthesis, FA elongation, FA degradation, steroid synthesis, arachidonic acid, and ether ester metabolism (Figure 8).

After further analysis of the functional assignments, seven and five genes were found to be responsible for the categories of glycerolipid metabolism and glycerophospholipid metabolism, respectively, among the 33 genes (Supplementary File 7). According to gene co-expression analysis, 13 genes were associated with FA biosynthesis and metabolism, of which three genes (*FAD2*, *LOC105174578*, and *LOC105169658*) regulated the biosynthesis of UFAs (Figure 8). In addition, four miRNAs (*osa-miR167a-5p_2ss2GA21AG*, *osa-miR167a-5p_2ss14AG21A*, *osa-miR167a-5p_2ss2GA21AT*, and *osa-miR167a-5p*) had the same target gene *LOC10514358* involved in FA elongation (Figure 8). In the gene co-expression network, we found that miRNA (*bol-MIR9410-p3_2ss4TG17TA*) could simultaneously regulate three target genes (*LOC105176298*, *LOC105167408*, and *LOC105158444*) involved in glycerophospholipid metabolism, FA biosynthesis, and FA degradation, respectively (Figure 8). Similarly, some important miRNAs and their target genes involved in glyceride metabolism, ether ester metabolism, arachidonic acid, and steroid synthesis have been linked. Notably, the data suggest that the coexpression network of miRNA and target genes can be used in further studies to identify the function of known and novel genes related to lipid and FA biosynthesis in sesame seed development.

## Discussion

To improve the varietal characteristics of sesame, increasing the UFA content (especially oil yield and FA composition) is the first and most important priority for the development of sesame breeding (Wang D.D. et al., 2018; Ahmed et al., 2020). With the possibility of the presence of the sesame genome and the advent of high-throughput sequencing for small RNA analysis in sesame seeds, we hereby elaborated the scope of transcriptomic, miRNA, degradation group analysis data, and gene network data during sesame seed development (Zheng et al., 2019).

In this study, 32 miRNA and target pairs involved in lipid and FA biosynthesis were screened out. Importantly, most of these target pairs were related to glyceride metabolism and FA biosynthesis, and five miRNA target gene pairs related to glycerol phospholipid metabolism were also identified by degradome sequencing data. Interestingly, seven target genes involved in FA degradation were found, but three of them did not have miRNA pairs. We further analyzed and found that the expression levels of four miRNA genes changed significantly with the maturity of sesame, such as, *stu-MIR8005c-p3_1ss13AG/LOC105159459* involved in glyceride metabolism, *sly-miR166c-5p_1ss21AG/FAD2* involved in UFA metabolism, *gma-MIR169o-p3_2ss16AC21CG/LOC105161564* involved in FA biosynthesis, and *PC-3p-62_86681/LOC105162196* involved in steroid synthesis. Thus, these four miRNA-target pairs were identified as potential regulators of the lipid and FA biosynthesis process during sesame seed development. To the knowledge of the authors, this is the first study ever to report novel miRNAs targeting miRNA-target pairs that had not been previously discovered, for example, *PC-3p-14226_618*, *PC-5p-228878_56*, *PC-5p-145800_91*, and *PC-3p-59282_203*. The results suggest that these specific miRNA target pairs may be involved in the regulatory network of lipid and FA synthesis, and play a central role in controlling gene expression and metabolic pathways throughout development periods.

**FIGURE 9:**
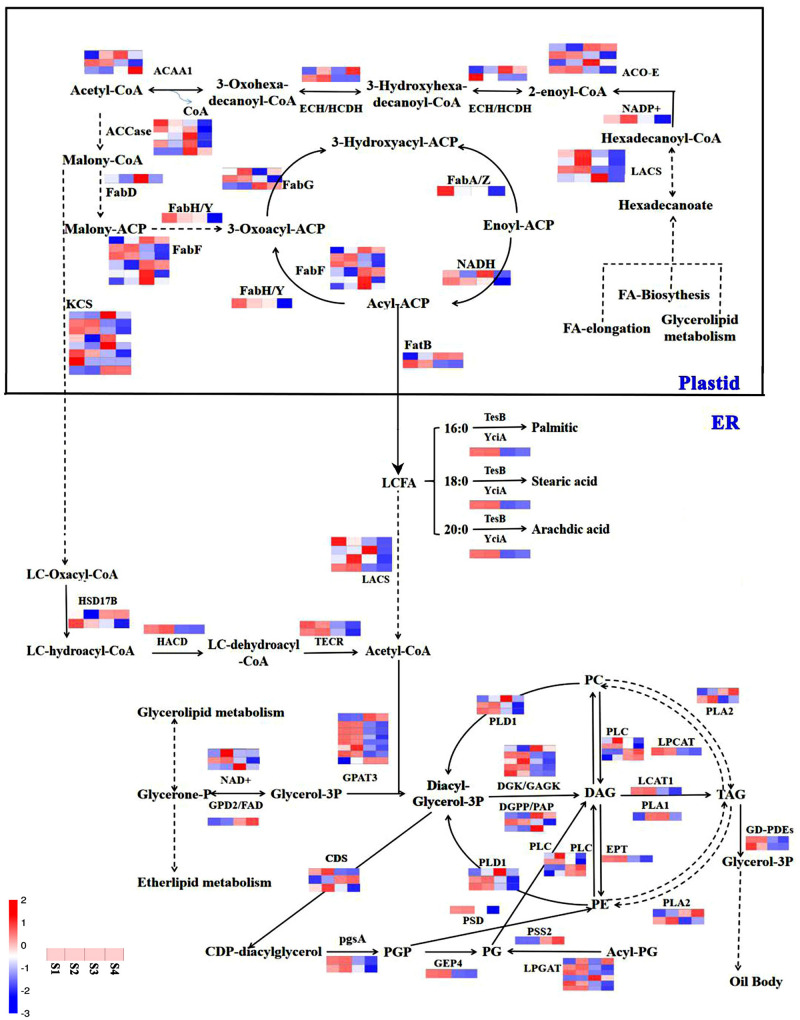
Relation network of miRNAs and target genes with lipid and fatty acid biosynthesis in sesame seeds. The blue color represents the down-regulated gene expression pattern, while the red color represents the up-regulated gene expression pattern.

Previous studies have shown that oil synthesis regulates *de novo* FA biosynthesis in the plastid and TAG assembly in the endoplasmic reticulum (ER), and that oil body formation (Reynolds et al., 2019) in oil plants is represented (Zheng et al., 2019). This study signified the presence of 106 unigenes involved in oil biosynthesis pathways (Figure 9). In general, lipid and FA syntheses are initiated with the supply of acetyl-CoA substrate (Wang et al., 2019), which is first converted to malonyl-CoA (Chen et al., 2020), and carbon flux is initiated *via* FA synthase and most other enzymes associated with lipid and FA biosynthesis (Huang, 1996). In this study, the initiation and acyl chain elongation of *de novo* FA biosynthesis were reported to be possibly regulated by 50 unigenes, and the latter showed apparent down-regulation of at least one isoform in S4 compared with S1, suggesting their functional involvement in the initiation of FA biosynthesis (Figure 9). In this study, ACCase catalyzed the first reaction involving four genes (conversion of acetyl-CoA to malonyl-CoA) involved in the biosynthesis of FA (Figure 9). Furthermore, FATA is highly specific for 18:1-ACP, and FATB is specific for acyl-A, which are known to regulate chain termination during FA synthesis (Lu et al., 2009). Unigenes *LOC105158149* and *LOC105160370*, which encode FATB, were up-regulated more than 10-fold during the S1 to S4 phase. In addition, four unigenes encoding LACS generating the acyl-CoA pool were detected, all of which were significantly down-regulated (Figure 9). The free long-chain FAs (16:0, 18:0, and 20:0) are esterified by LACS and output to ER for further acyl processing (Bates et al., 2009). Pathways such as glycerolipid metabolism and ether-lipid metabolism are known to be linked by the compound, which converts to glycerone-3P (G3P) under the catalyzation of *NAD* + and *FAD2* (Hu et al., 2009; Dehghan and Yarizade, 2014; Yasemin et al., 2018; Macovei et al., 2021). 49 unigenes related to the formation of TAG were identified, e.g., long-chain acyl-CoA and G3P, which could start the assemble of TAG; diacylglycerol acyltransferase (DGAT), which was the major enzymes that catalyze diacylglycerol (DAG); and phosphatidylcholine (an acyl donor), which could combine with oleosin to form an oil body (Kennedy, 1961; Peng and Weselake, 2011). Most of the unigenes encoding DAG were reported with high transcript levels, suggesting that they are an important signaling pathway during sesame seed development (Wang R.K. et al., 2018; Huai et al., 2020). On the other hand, relatively active unigenes, encoding acyl editing, PE-DAG, and PC-DAG interconversion were likely responsible for the incorporation of UFAs into TAG in sesame seeds (Peng and Weselake, 2011; Wang R.K. et al., 2018; Yang et al., 2018; Tyagi et al., 2019). The data emphasized the essential roles of *FAD2*, FabF/KASII, LACS, ACO-E, LPCAT, and some unknown genes during the biosynthesis of FA and other UFAs during sesame seed development (Figure 9).

Overall, the regulatory co-expression network construction data showed that the nine kinds of oil synthesis related pathways were the most likely regulatory factors of lipid and FA biosynthesis; and so far, it can be stated that miRNA regulates the complex network of genes and their target genes involved in various physiological and biological functions such as regulation of sesame responses to oil synthesis. These target pairs *gma-MIR1510a-p3_2ss12AT19CT/LOC105159459*, *sly-miR166c-5p_1ss21AG/FAD2*, *gma-MIR169o-p3_2ss16AC21CG/LOC10516 1564*, and *PC-3p-62_86681/LOC105162196* were found to be potentially relevant to oil synthesis in developing sesame seeds. Nevertheless, future investigation of these four pairs is warranted to reveal their precise molecular functions and regulatory mechanisms. Herein, complementary comparative multi-omics data were generated in four successive developmental periods to unravel the important pathways responsible for the high-oil yield and increased UFA content in sesame seeds. The results collected would also fill the knowledge gap on the mechanism of oil deposition and FA saturation transformation during embryonic development in other oil plant seeds.

## Conclusion

In conclusion, this study provides a comprehensive framework for a better understanding of the lipid and FA biosynthesis mechanism of sesame under different growth periods. The1,006 target genes of 13,801 miRNAs were extracted from mixed degradome sequencing, which accounted for 65 novel miRNAs targeting 8,051 transcripts, and 3,705 target genes of 220 miRNAs were also differentially manifested. At least 12 potential lipid-related miRNAs and target genes were analyzed by RNA-seq and qRT-PCR. Perfect harmonization of high level of *FAD2* in the mature period promoted the increase of linoleic acid content in sesame. Thirty-three co-expressed genes formed a co-regulatory sub-network, which showed that some important miRNAs and their target genes were involved in the pathways of lipid and FA biosynthesis such as glyceride metabolism, ether ester metabolism, arachidonic acid, and steroid synthesis, and the pathways were also found to be interconnected. In addition, the biosynthetic and regulatory genes identified from the genome-wide co-expression network may expand the understanding of lipid and FA biosynthesis in other plant seeds and increase the functional value of the resulting food products.

## Data Availability Statement

The datasets presented in this study can be found in online repositories. The names of the repository/repositories and accession number(s) can be found below: https://www.ncbi.nlm.nih.gov/bioproject/?term=PRJNA739094.

## Author Contributions

Y-PZ: conceptualization, methodology, software, and writing–original draft preparation. Y-YZ: data curation and validation. KT: data curation and investigation. FZ and J-GZ: methodology and software. FH: visualization and investigation. P-CW: visualization, supervision, and investigation. Z-JW: funding acquisition, supervision, and writing–reviewing and editing. All authors contributed to the article and approved the submitted version.

## Conflict of Interest

The authors declare that the research was conducted in the absence of any commercial or financial relationships that could be construed as a potential conflict of interest.

## Publisher’s Note

All claims expressed in this article are solely those of the authors and do not necessarily represent those of their affiliated organizations, or those of the publisher, the editors and the reviewers. Any product that may be evaluated in this article, or claim that may be made by its manufacturer, is not guaranteed or endorsed by the publisher.

## Footnotes

1
http://genome.ucsc.edu/

2
https://www.ncbi.nlm.nih.gov/genome/?term=Sesamum+indicum+L

3
ftp://mirbase.org/pub/mirbase/CURRENT/

## Supplementary Material

Supplementary Figure 1 Heatmap of potential DEmiRNA significantly expressed across the four stages with similar distribution patterns. The blue color represents the down-regulated gene expression pattern, while the red color represents the up-regulated gene expression pattern.

Supplementary Figure 2 GO enrichment of genes significantly expressed across the four stages.

Supplementary File 1 Sequences and primers of miRNAs and their target genes in RT-qPCR test experiment.

Supplementary File 2 Length distribution of unique miRNAs.

Supplementary File 3 miRNAs were categorized into five different groups based on the abundance of miRNAs expression levels.

Supplementary File 4 Analysis of miRNA first nucleotide bias total.

Supplementary File 5 Sequencing result of identified miRNAs and their targets genes during sesame seed development.

Supplementary File 6 Identify of miRNAs and transcript targets from mixed degradome data.

Supplementary File 7 Co-expression network involved genes of nine categories.

## References

[B1] Adebowale A. A. Sanni S. A. Falore O. A. (2011). Varietal differencesin the physical properties and proximate. *World J. Agric. Sci.* 7 42–46.

[B2] Ahmed I. A. M. Aljuhaimi F. Özcan M. M. Ghafoor K. Şimşek Ş Babiker E. E. (2020). Evaluation of chemical properties, amino acid contents and fatty acid compositions of sesame seed provided from different locations. *J. Oleo Sci.* 69 795–800. 10.5650/jos.ess20041 32641612

[B3] Bates P. D. Durrett T. P. Ohlrogge J. B. Pollard M. (2009). Analysis of acyl fluxes through multiple pathways of triacylglycerol synthesis in developing soybean embryos. *Plant Physiol.* 150 55–72. 10.1104/pp.109.137737 19329563 PMC2675710

[B4] Bhunia R. K. Chakraborty A. Kaur R. Gayatri T. Bhat K. V. Basu A. (2015). Analysis of fatty acid and lignan composition of Indian germplasm of sesame to evaluate their nutritional merits. *J. Am. Oil Chem. Soc.* 92 65–76. 10.1007/s11746-014-2566-3

[B5] Bisgin H. Gong B. S. Wang Y. P. Tong W. D. (2018). Evaluation of bioinformatics approaches for next-generation sequencing analysis of microRNAs with a toxicogenomics study design. *Front. Genet.* 9:22. 10.3389/fgene.2018.00022 29467792 PMC5808213

[B6] Chen D. J. Luo X. G. Yan L. H. Si C. L. Wang N. He H. P. (2020). Transcriptome analysis of unsaturated fatty acids biosynthesis shows essential genes in sprouting of *Acer truncatum* Bunge seeds. *Food Biosci.* 2020:100739. 10.1016/j.fbio.2020.100739

[B7] Dan M. Huang M. H. Liao F. Qin R. Y. Liang X. J. Zhang E. Z. (2018). Identification of ethylene responsive miRNAs and their targets from newly harvested banana fruits using high-throughput sequencing. *J. Agric. Food Chem.* 66 10628–10639. 10.1021/acs.jafc.8b01844 30192539

[B8] Dbrowski G. Czaplicki S. Konopka I. (2020). Composition and quality of poppy (*Papaversomniferum* L.) seed oil depending on the extraction method. *LWT Food Sci. Technol.* 134:110167. 10.1016/j.lwt.2020.110167

[B9] Dehghan N. F. Yarizade K. (2014). Bioinformatics study of delta-12 fatty acid desaturase 2 (FAD2) gene in oilseeds. *Mol. Biol. Rep.* 41 5077–5087. 10.1007/s11033-014-3373-5 24816719

[B10] Dossa K. Diouf D. Wang L. Wei X. Zhang Y. Niang M. (2017). The emerging oilseed crop sesamum indicum enters the “Omics” Era. *Front. Plant Sci.* 8:1154. 10.3389/fpls.2017.01154 28713412 PMC5492763

[B11] Elleuch M. Besbes S. Roiseux O. Blecker C. Attia H. (2007). Quality characteristics of sesame seeds and byproducts. *Food Chem.* 103 641–650. 10.1016/j.foodchem.2006.09.008

[B12] Gacek K. Bayer P. E. Bartkowiak-Broda I. Szala L. Bocianowski J. Edwards D. (2017). Genome-wide association study of genetic control of seed fatty acid biosynthesis in *Brassica napus*. *Front. Plant Sci.* 7:2062. 10.3389/fpls.2016.02062 28163710 PMC5247464

[B13] Han X. J. Yin H. F. Song X. X. Zhang Y. X. Liu M. Y. Sang J. (2016). Integration of small RNAs, degradome and transcriptome sequencing in hyperaccumulator Sedum alfredii uncovers a complex regulatory network and provides insights into cadmium phytoremediation. *Plant Biotechnol. J.* 14 1470–1483. 10.1111/pbi.12512 26801211 PMC5066797

[B14] Hu Y. P. Wu G. Cao Y. L. Wu Y. H. Xiao L. Li X. D. (2009). Breeding response of transcript profiling in developing seeds of *Brassica napus*. *BMC Mol. Biol.* 10:49. 10.1186/1471-2199-10-49 19463193 PMC2697984

[B15] Huai D. X. Xue X. M. Li Y. Wang P. Li J. G. Yan L. Y. (2020). Genome-wide identification of peanut KCS genes reveals that AhKCS1 and AhKCS28 are involved in regulating VLCFA contents in seeds. *Front. Plant Sci.* 11:406. 10.3389/fpls.2020.00406 32457765 PMC7221192

[B16] Huang A. (1996). Oleosins and oil bodies in seeds and other organs. *Plant Physiol.* 110:1055. 10.1104/pp.110.4.1055 8934621 PMC160879

[B17] Huang J. Q. Zhang T. Zhang Q. X. Chen M. Wang Z. J. Zheng B. S. (2016). The mechanism of high contents of oil and oleic acid revealed by transcriptomic and lipidomic analysis during embryogenesis in Carya cathayensisSarg. *BMC Genom.* 17:113. 10.1186/s12864-016-2434-7 26878846 PMC4755018

[B18] Huang R. M. Zhou Y. Zhang J. P. Ji F. Y. Jin F. Fan W. (2021). Transcriptome analysis of walnut (*Juglans regia* L.) embryos reveals key developmental stages and genes involved in lipid biosynthesis and polyunsaturated fatty acid metabolism. *Agric. Food Chem.* 69 377–396. 10.1021/acs.jafc.0c05598 33373225

[B19] Kennedy E. P. (1961). Biosynthesis of complex lipids. *Fed Proc.* 20:934. 14455159

[B20] Kumar A. P. K. McKeown P. C. Boualem A. Ryder P. Brychkova G. Bendahmane A. (2017). Tilling by Sequencing (TbyS) for targeted genome mutagenesis in crops. *Mol. Breed.* 37:14.

[B21] La Sala L. Micheloni S. De Nigris V. Prattichizzo F. Ceriello A. (2018). Novel insights into the regulation of miRNA transcriptionalcontrol: implications for T2D and related complications. *Acta Diabetol.* 1:10. 10.1007/s00592-018-1149-4 29732466

[B22] Lei P. Liu Z. Hu Y. B. Kim H. C. Liu S. Liu J. Q. (2021). Transcriptome analysis of salt stress responsiveness in the seedlings of wild and cultivated *ricinus communis* L. *J. Biotechnol.* 327 106–116. 10.1016/j.jbiotec.2020.12.020 33421510

[B23] Li S. S. Wang L. S. Shu Q. Y. Wu J. Chen L. G. Shao S. (2015). Fatty acid composition of developing tree peony (Paeonia section Moutan DC.) seeds and transcriptome analysis during seed development. *BMC Genom.* 16:208. 10.1186/s12864-015-1429-0 25887415 PMC4404109

[B24] Liu H. Y. Tan M. P. Yu H. J. Li L. Zhou F. Yang M. M. (2016). Comparative transcriptome profiling of the fertile and sterile flower buds of a dominant genic male sterile line in sesame (*Sesamum indicum* L.). *BMC Plant Biol.* 16:250. 10.1186/s12870-016-0934-x 27832742 PMC5105256

[B25] Lu C. F. Xin Z. G. Ren Z. H. Miquel M. Browse J. (2009). An enzyme regulating triacylglycerol composition is encoded by the ROD1 gene of *Arabidopsis*. *Proc. Natl. Acad. Sci. U.S.A.* 106 18837–18842. 10.1073/pnas.0908848106 19833868 PMC2774007

[B26] Lyu Y. S. Wei X. J. Zhong M. Niu S. Ahmad S. Shao G. N. (2020). Integrated transcriptome, small rna, and degradome analysis to elucidate the regulation of rice seedling mesocotyl development during the passage from darkness to light. *Crop J.* 8 44–54.

[B27] Macovei A. Rubio-Somoza I. Paiva J. A. P. Araújo S. Donà M. (2021). Editorial: MicroRNA signatures in plant genome stability and genotoxic stress. *Front. Plant Sci.* 12:683302. 10.3389/fpls.2021.683302 33968124 PMC8100575

[B28] Majdalawieh A. F. Dalibalta S. Yousef S. M. (2020). Effects of sesamin on fatty acid and cholesterol metabolism, macrophage cholesterol homeostasis and serum lipid profile: a comprehensive review. *J. Eur J Pharmacol.* 885:173417. 10.1016/j.ejphar.2020.173417 32750369

[B29] Peng F. Y. Weselake R. J. (2011). Gene coexpression clusters and putative regulatory elements underlying seed storage reserve accumulation in *Arabidopsis*. *BMC Genom.* 12:286. 10.1186/1471-2164-12-286 21635767 PMC3126783

[B30] Reynolds K. B. Cullerne D. P. Anna E. T. Rolland V. Blanchard C. L. Wood C. C. (2019). Identification of genes involved in lipid biosynthesis through de novo transcriptome assembly from cocos nucifera developing endosperm. *Plant Cell Physiol.* 60 945–960. 10.1093/pcp/pcy247 30608545 PMC6498750

[B31] Sevgi M. (2018). Identification and functional analyses of new sesame miRNAs (*Sesamum indicum* L.) and their targets. *Mol. Biol. Rep.* 45 2145–2155. 10.1007/s11033-018-4373-7 30209739

[B32] Sui N. Wang Y. Liu S. Yang Z. Wang F. Wan S. (2018). Transcriptomic and physiological evidence for the relationship between unsaturated fatty acid and salt stress in peanut. *Front. Plant Sci.* 9:7. 10.3389/fpls.2018.00007 29403517 PMC5786550

[B33] Tyagi S. Sri T. Singh A. Mayee P. Shivaraj S. M. Sharma P. (2019). Suppressor of overexpression of CONSTANS1 influences flowering time, lateral branching, oil quality, and seed yield in *Brassica juncea* cv. *Varuna. FunctIntegr. Genomic.* 19 43–60. 10.1007/s10142-018-0626-8 29943206

[B34] Wacal C. Ogata N. Basalirwa D. Sasagawa D. Kato M. Handa T. (2019). Nishihara, E. Fatty acid composition of sesame (*Sesamum indicum* L.) seeds in relation to yield and soil chemical properties on continuously monocropped upland fields converted from paddy fields. *Agron* 9:801. 10.3390/agronomy9120801

[B35] Wang D. D. Zhang L. X. Huang X. R. Wang X. Yang R. N. Mao J. (2018). Identification of nutritional components in black sesame determined by widely targeted metabolomics and traditional chinese medicines. *Molecules* 23:1180. 10.3390/molecules23051180 29762486 PMC6100530

[B36] Wang R. K. Liu P. Fan J. S. Li L. L. (2018). Comparative transcriptome analysis two genotypes of Acer truncatum Bunge seeds reveals candidate genes that influences seed VLCFAs accumulation. *Sci. Rep.* 8:15504. 10.1038/s41598-018-33999-3 PMC6195533 30341360

[B37] Wang L. H. Li D. H. Zhang Y. X. Gao Y. Yu J. Y. Wei X. (2016). Tolerant and susceptible sesame genotypes reveal waterlogging stress response patterns. *PLoS One* 11:e0149912. 10.1371/journal.pone.0149912 26934874 PMC4774966

[B38] Wang Q. Zhao L. Wang B. C. Cao W. X. Xu G. Z. Chen P. (2011). Breeding and high yield cultivation techniques of a New Sesame Variety Wanzhi 2 with high quality. *Anhui Agron. Bull.* 17 62–64.

[B39] Wang X. J. Liang H. Y. Guo D. L. Guo L. L. Duan X. G. Jia Q. S. (2019). Integrated analysis of transcriptomic and proteomic data from tree peony (*P. ostii*) seeds reveals key developmental stages and candidate genes related to oil biosynthesis and fatty acid metabolism. *Hortic. Res.* 6:111. 10.1038/s41438-019-0194-7 PMC6804530 31645965

[B40] Wei L. B. Zhang H. Y. Duan Y. H. Li C. Chang S. X. Miao H. M. (2016). Transcriptome comparison of resistant and susceptible sesame (*Sesamum indicum* L.) varieties inoculated withFusarium oxysporum f. sp. sesami. *Plant Breed.* 135 627–635. 10.1111/pbr.12393

[B41] Wei X. Zhu X. D. Yu J. Y. Wang L. H. Zhang Y. X. Li D. H. (2016). Identification ofsesame genomic variations from genome comparison of landrace and variety. *Front. Plant Sci.* 7:1169. 10.3389/fpls.2016.01169 27536315 PMC4971434

[B42] Xu X. B. Yin L. L. Ying Q. C. Song H. M. Xue D. W. Lai T. F. (2013). High-throughputsequencing and degradome analysis identify miRNAs and theirtargets involved in fruit senescence of *Fragaria ananassa*. *PLoS One* 8:e70959. 10.1371/journal.pone.0070959 23990918 PMC3747199

[B43] Yan R. Liang C. Z. Meng Z. G. Malik W. Zhu T. Zong X. F. (2016). Progress in genomesequencing will accelerate molecular breeding in cotton (*Gossypium* spp.). *3 Biotech.* 6:217. 10.1007/s13205-016-0534-3 PMC5055485 28330289

[B44] Yang T. Q. Yu Q. Xu W. Li D. Z. Chen F. Liu A. Z. (2018). Transcriptome analysis reveals crucial genes involved in the biosynthesis of nervonic acid in woody *Malania oleifera* oilseeds. *BMC Plant Biol.* 18:247. 10.1186/s12870-018-1463-6 30340521 PMC6195686

[B45] Yanik H. Turktas M. Dundar E. Hernandez P. Dorado G. Unver T. (2013). Genome-wide identification of alternate bearing-associatedmicroRNAs (miRNAs) in olive (*Olea europaea* L). *BMC Plant Biol.* 13:10. 10.1186/1471-2229-13-10 23320600 PMC3564680

[B46] Yasemin C. A. Mehmet U. N. Cengiz B. M. Ulu F. Can T. F. Cetinkaya R. (2018). Comparative identification and evolutionary relationship of fatty acid desaturase (FAD) genes in some oil crops: the sunflower model for evaluation of gene expression pattern under drought stress. *Biotechnol. Biotec. Eq.* 32 846–857. 10.1080/13102818.2018.1480421

[B47] Yin D. D. Li S. S. Shu Q. Y. Gua Z. Y. Wu Q. Feng C. Y. (2018). Identification of microRNAs and long non-coding RNAs involved in fatty acid biosynthesis in tree peony seeds. *Gene* 666 72–82. 10.1016/j.gene.2018.05.011 29738839

[B48] Yuan C. Wang X. L. Geng R. Q. He X. L. Qu L. Chen Y. L. (2013). Discovery of cashmere goat (*Capra hircus*) microRNAs in skin and hair follicles by Solexa sequencing. *BMC Genom.* 14:511. 10.1186/1471-2164-14-511 23889850 PMC3765263

[B49] Zhang Y. J. Gong H. H. Li D. H. Zhou R. Zhao F. T. Zhang X. R. (2020). Integrated small RNA and Degradome sequencing provide insights into salt tolerance in sesame (*Sesamum indicum* L.). *BMC Genom.* 21:494. 10.1186/s12864-020-06913-3 32682396 PMC7368703

[B50] Zhang Y. J. Zhang Q. Y. Weng X. C. Du Y. H. Zhou X. (2021). NEase-based amplification for detection of miRNA, multiple miRNAs and circRNA. *Anal. Chim. Acta* 1145 52–58. 10.1016/j.aca.2020.12.024 33453881

[B51] Zhao S. Q. Mi X. Z. Guo R. Xia X. B. Liu L. An Y. L. (2020). The biosynthesis of main taste compounds is coordinately regulated by miRNAs and phytohormones in tea plant (*Camellia sinensis*). *J. Agric. Food Chem.* 68 6221–6236. 10.1021/acs.jafc.0c01833 32379968

[B52] Zhao S. Q. Wang X. W. Yan X. M. Guo L. X. Mi X. Z. Xu Q. S. (2018). Revealing of the microRNA involved regulatory gene networks on terpenoid biosynthesis in *Camellia sinensis* in different growing time points. *J. Agric. Food Chem.* 66 12604–12616. 10.1021/acs.jafc.8b05345 30400742

[B53] Zheng Y. S. Chen C. J. Liang Y. X. Sun R. H. Gao L. C. Liu T. (2019). Genome-wide association analysis of the lipid and fatty acid metabolism regulatory network in the mesocarp of oil palm (*Elaeisguineensis* Jacq.) based on small noncoding RNA sequencing. *Tree Physiol.* 39 356–371. 10.1093/treephys/tpy091 30137626

[B54] Zhu J. F. Zhang M. M. Guo J. X. Wu X. K. Wang S. M. (2021). Metabolite profiling of chondrosarcoma cells: a robust gc-ms method for the analysis of endogenous metabolome. *J. Chromatogr. B* 1169:122606. 10.1016/j.jchromb.2021.122606 33684880

