# Supplementary figures and images for: Integration of miRNAs, Degradome, and Transcriptome Omics Uncovers a Complex Regulatory Network and Provides Insights Into Lipid and Fatty Acid Synthesis During Sesame Seed Development
Source: Front Plant Sci. 2021 Jul 29;12:709197. doi: 10.3389/fpls.2021.709197 (PMC8358462; doi:10.3389/fpls.2021.709197)

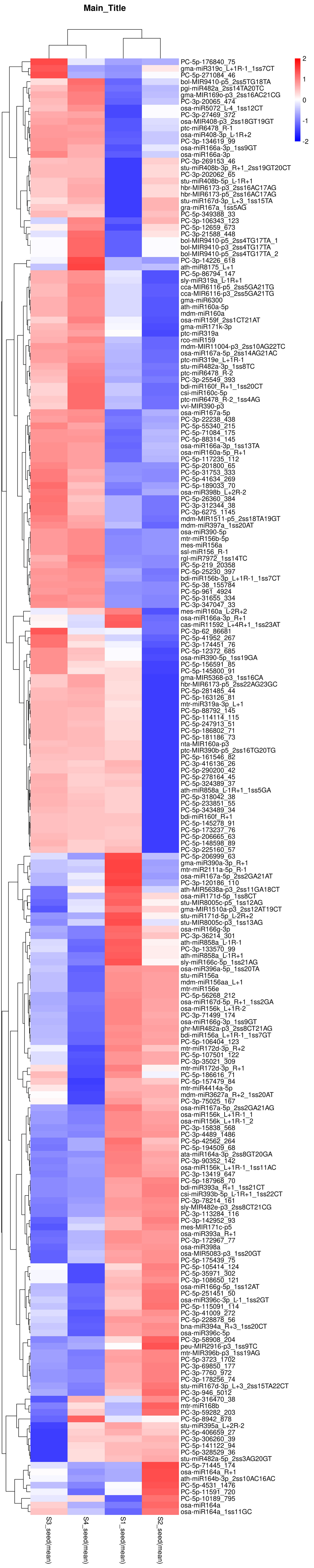

Supplement: Supplementary Figure 1 — Heatmap of potential DEmiRNA significantly expressed across the four stages with similar distribution patterns. The blue color represents the down-regulated gene expression pattern, while the red color represents the up-regulated gene expression pattern. [file Image_1.JPEG]
